# Zinc Carboxylate Surface Passivation for Enhanced Optical Properties of In(Zn)P Colloidal Quantum Dots

**DOI:** 10.3390/mi13101775

**Published:** 2022-10-19

**Authors:** Doheon Yoo, Eunyoung Bak, Hae Mee Ju, Yoo Min Shin, Min-Jae Choi

**Affiliations:** Department of Chemical and Biochemical Engineering, Dongguk University, Pildong-ro 1-gil, Jung-gu, Seoul 04620, Korea

**Keywords:** indium phosphide, surface passivation, colloidal quantum dots, photoluminescence

## Abstract

Indium phosphide (InP) colloidal quantum dots (CQDs) have generated great interest as next-generation light-emitting materials owing to their narrow emission spectra and environment-friendly components. The minimized surface defects is essential to achieve narrow full-width at half-maximum (FWHM) and high photoluminescence quantum yield (PLQY). However, InP CQDs are readily oxidized in ambient condition, which results in formation of oxidation defect states on the surface of InP CQDs. Herein, we introduce a strategy to successfully passivate the surface defects of InP core by zinc complexes. The zinc carboxylates passivation reduces FWHM of InP CQDs from 130 nm to 70 nm and increases PLQY from 1% to 14% without shelling. Furthermore, the photoluminescence (PL) peak has shifted from 670 nm to 510 nm with an increase of zinc carboxylates passivation, which suggests that excessive zinc carboxylates functions as a size-regulating reagent in the synthesis.

## 1. Introduction

Colloidal quantum dots (CQDs) are promising semiconducting nanomaterials for which the optoelectronic properties can be controlled by tuning their physical size [1,2,3,4]. Owing to their tunable optical characteristics, CQDs have been attracted in several applications such as energy harvesting [5,6,7], infrared imaging [8,9], and photocatalysts [10,11]. Moreover, their colloidal stability enables to be employed in solution-processed fabrication methods such as inkjet-printing [12,13,14], for the integration in next-generation optoelectronic devices including display devices [15,16,17].

To avoid restriction of hazardous substances (RoHS) directive, III-V CQDs such as indium phosphide (InP) [15,18,19], and indium arsenide (InAs) [18,19] have been recently attracted as candidates to replace traditional Cd, Pb-based CQDs [2,4]. However, those III-V CQDs are readily oxidized to form oxidation states, which generates trap states. These trap states result in non-radiative recombination and deteriorates the optical properties of the III-V QD [20,21,22,23]. Various methods such as metal doping (Cu, Ag, Mn) and halogenic ions have been used to prevent formation of surface trap states, including the further shell passivation process [24,25,26].

Another way to prevent surface oxidation of CQDs is adding additional surface ligands for conformal surface passivate. For InP CQDs, it was reported that zinc complexes form In(Zn)P outer layer that protects the InP core from oxidation even after the washing and redispersion process [27,28]. However, the quantitative analyses on function of zinc complexes in InP CQDs are not fully explores yet.

Here we study the effect of zinc carboxylate surface passivation on InP and InP@ZnS CQDs. By controlling the ratio between zinc carboxylate and indium acetate, we systematically observe the relationship between loading amounts of zinc carboxylates and optical properties of InP CQDs. When the Zn/In ratio is ~3.2, the InP CQDs exhibits the highest photoluminescence quantum yield (PLQY) of 14%, which is an almost 14-fold increase compared to the InP CQDs without zinc carboxylate passivation. By introducing a thin ZnS outer shell, the PLQY of In(Zn)P CQDs further increase to 21%.

Furthermore, we find that zinc carboxylate complex delays the early-state depletion of phosphine precursor, which can modulate the growth of InP core. This leads to a decrease of InP cores size by 18%, which was confirmed by photoluminescence (PL) measurement and TEM analysis. The delayed phosphine precursor depletion results in uniform size distribution of InP core, which reduces full-width at half-maximum (FWHM) from 130 to 70 nm, sharpening the emission spectrum.

## 2. Materials and Methods

### 2.1. Materials

All the chemicals were purchased from Sigma-Aldrich and used without further purification. zinc acetate (Zn(Ac)_2_ 99%), indium acetate (In(Ac)_3_ 99.99%), myristic acid (MA, 99%), 1-octadecene (ODE, >98%), oleic ocid(OA, 98%), 1-dodecanethiol (1-DDT, >98%), trimethylsilyl phosphine ((TMS)_3_P, 99%)

### 2.2. Synthesis of Zinc Oleate (Zn(OA)_2_) Stock Solution

A 25 mmol of zinc acetate, 55 mmol of OA and 25 mL of ODE were incorporated into a 250 mL three-necked round bottom flask. The solution was degassed for 8 h at 120 °C. The solution was cooled down to ambient temperature after the yellowish solution of Zn (OA)_2_ was obtained, which was stored in the glove box for further use.

### 2.3. Synthesis of In(Zn)P QDs

A In(Ac)_3_ (0.1 mmol), Zn(Ac)_2_ (0.4166 mmol; 1 eq. of Zinc per 0.1 mmol of In(Ac)_3_), MA(0.9333 mmol) and 10 mL of ODE was put into a 50 mL round-bottom three-neck flask. The solution was degassed at 110 °C for 1 hr. After degassing, the solution was heated up to 245 °C under an inert atmosphere, and then 0.75 mmol (TMS)_3_P was rapidly injected into the solution. The In(Zn)P core was grown for 10 min. After finishing the core growth, the solution was rapidly quenched to room temperature with compressed air and an ice bath.

Synthesized In(Zn)P core has been washed twice with acetone and ethyl alcohol mixture and precipitated through centrifugation. To filter out the remaining, unreacted zinc carboxylate residue, the solution was filtered once with a 0.22 μm syringe filter. Purified QDs were redispersed to toluene for further characterization. For the case of cores prepared for shell passivation, it was purified only once and redispersed to hexane for further use.

### 2.4. ZnS Shell Passivaiton to In(Zn)P QDs

For the passivation of the ZnS shell, 0.1 mmol of In(Zn)P QD solution previously dispersed to hexane, Zn(OA)_2_ (0.4166 mmol), 1-DDT (0.4166 mmol), and 5 mL of ODE were incorporated into a three-neck round-bottom flask. The solution was degassed at 110 °C for 2 h. After degassing, the solution was heated up to 300 °C and kept for 20 min for the passivation of ZnS shell. The solution was rapidly quenched to room temperature with compressed air and an ice bath to conclude the reaction.

The core was washed and precipitated in the same manner as In(Zn)P core, then redispersed to toluene for further characterization.

### 2.5. Characterization Methods

Photoluminescence (PL) spectroscopy and QY were measured using a Horiba Fluromax+ spectrofluorometer (Horiba Scientific, Kyoto, Japan), Uv-Vis. Spectroscopy was conducted by Jasco V-770 Spectrophotometer (Jasco, Kawasaki, Japan). X-ray photoelectron spectroscopy (XPS) analysis was conducted through Versaprobe II (ULVAC-PHI, Chigasaki, Japan). For XPS analysis, the In(Zn)P QDs were spin-cast onto the Si wafer. PL spectroscopy, QY measurement, UV-Vis. Spectroscopy and XPS analysis was conducted at Colloids & Nanomaterials Analysis Center, Dongguk University, Seoul, South Korea. TEM image was taken using JEOL JEM-2100F HR (JEOL, Tokyo, Japan)at the Korea Advanced Institute of Science (KAIST), National Nanofab Center (NNFC), Daejeon, South Korea. For the case of the TEM analysis, the samples were dispersed in cyclohexene after a single washing.

## 3. Results and Discussions

As mentioned above, the formation of a surficial oxidative layer upon the core causes critical deterioration of the optical properties of III-V CQD. To maintain the optical performances of the core, passivation of the InP core with zinc carboxylate can direct to the prevention of the In_2_O_3_ layer which may greatly aggravate the optical characteristics such as PLQY and FWHM of the synthesized core.

To prevent the development of the oxide layer on the surface of the InP core, several strategies can be adopted, such as the utilization of ligands with long alkyl chains, or epitaxially growing the heterogenous II-VI material layer onto the core to form a core-shell structure that passivates surficial defects [17,29,30]. Utilization of long alkyl chain ligands may prevent the degradation of the core; its effect is rather not prominent than passivating the additional shell layer. On the other aspect, passivation of the heterogeneous epitaxial layer onto the core may cause red-shifted PL spectrum [31,32] in exchange for an extraordinary increase in PLQY [3,29,30] as surficial lattice mismatch leads to strain-oriented defect between layers, generating a trap state.

To effectively modulate the optical properties of the InP core while retaining a reasonable level of optical properties, the utilization of the metal carboxylate complex as a temporal passivating agent can fulfill the requirements for the synthesis of the InP core with precisely controlled optical properties. In this research, we used the zinc myristate (Zn(MA)_2_) complex as a provisional passivating agent. As mentioned in the experimental section, we incorporated zinc acetate (Zn(Ac)_2_) with myristic acid (MA) with indium acetate (In(Ac)_3_) to induce the formation of the Zn(MA)_2_ complex during the degassing process.

For the Indium to Zinc ratio calculation, we considered 0.4166 mmol of zinc precursor as 1 equivalent per 0.1 mmol of indium precursor. For convenience, we marked each sample in the numerical manner of ‘Zn n’ (*n* = 0, 0.4, 0.8, 1.2) where the number *n* implies the amount of zinc precursor amplification rate compared to the original value of 0.4166 mmol. As demonstrated in the experimental section, the ratio of Zn(Ac)_2_ was carefully adjusted to find out the difference in optical properties upon the variance in the amount of synthesized zinc cluster.

**Figure 1:**
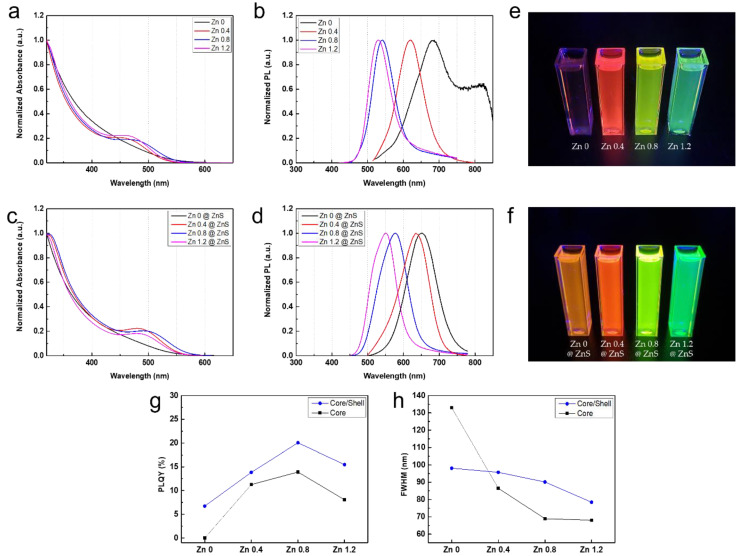
(**a**–**d**) absorption spectroscopy and PL measurement of In(Zn)P core (**a**,**b**) and In(Zn)P @ ZnS CQDs (**c**,**d**). Samples under UV illumination of In(Zn)P core (left to right, Zn 0, Zn 0.4, Zn 0.8, Zn1.2) (**e**) and In(Zn)P @ ZnS CQDs (left to right, Zn 0 @ ZnS, Zn 0.4 @ ZnS, Zn 0.8 @ ZnS, Zn 1.2 @ ZnS) (**f**). (**g**,**h**) PLQY (**g**) and FWHM (**h**) of CQDs depending on Zn loading amounts in the synthesis.

As shown in Figure 1a–d, the higher amount of the zinc complex during the core synthesis induced intensified blue-shift in the PL peak position and the 1 s excitonic peak of the absorption spectrum. For the case of the core, the shift between the sample passivated with 1.2 times zinc carboxylate and one without zinc passivation was observed to be ~100 nm. This trend in PL spectroscopy suggests that the zinc complex also decreased the average size of the synthesized core, hence zinc complex not only functions as a passivation medium but also acts as a size-controlling reagent. The size-controlling functionalities of zinc carboxylate complexes will be discussed in the following section. Apart from the size decrease caused by the utilization of the zinc carboxylate precursor, zinc alloying with InP matrix may also result in the blue shift of the PL spectrum. Kirkwood et al. reported extended X-ray absorbance fine structure spectroscopy (EXAFS) analysis, which suggests that zinc complexes tend to reside on the surface rather than forming an alloy [27].

As expected, all the In(Zn)P cores and cores with thin ZnS shell passivation showed narrowing FWHM with magnification in the zinc quantity, depicted in Figure 1h. However, the PLQY of synthesized samples was slightly diverted from the overall tendency, in which the highest PLQY of ~12% was achieved at Zn 0.8, then decreased to half when 1.2 times of zinc was used for the synthesis. This indicates that the optimal ratio of zinc carboxylate to indium precursor is about Zn 0.8, which is 0.333 mmol Zn(Ac)_2_ per 0.1 mmol of In(Ac)_3_.

Not only bounded to enhancement in photoluminescence properties of the core, but the zinc carboxylate also seems to possess a size-controlling ability of the QD core through interaction with phosphine precursor. The conventional (TMS)_3_P precursor is widely used in the synthesis of the III-V quantum dot, due to its extreme reactivity that would induce rapid and homogeneous nucleation, while its nature leads to instant depletion of (TMS)_3_P in the solution, inhibiting the diffusion-controlled growth processes [26,27], which is indispensable for the synthesis of monodisperse CQDs. However, under the presence of a zinc complex, the zinc complex creates a zinc-phosphine complex which would function as a continuous phosphine reservoir that would enable monodisperse growth through the diffusional growth mechanism [33].

**Figure 2:**
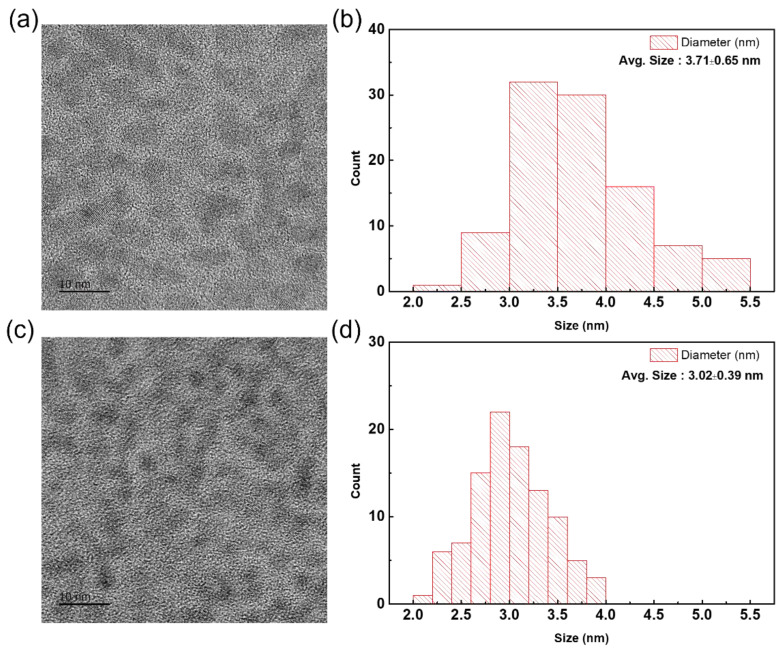
TEM images of synthesized In(Zn)P @ ZnS QDs and particle size distribution. (**a**,**b**) InP @ ZnS (Zn 0); (**c**,**d**) In(Zn)P @ ZnS (Zn 1.2).

In Figure 1a, from the two distinguishable PL spectrums of each sample—Zinc eq. 0.0 and Zinc eq. 1.2—the InP core synthesized without zinc complex showed heterogeneous growth in terms of the size distribution of the particle, while the sample with the maximum amount of zinc incorporated achieved monodisperse growth which can be induced from the like PL spectra which depicts shape assembled to Gaussian distribution. Furthermore, in the TEM images of Figure 2a,c the sample synthesized under the presence of zinc showed a much more uniform and smaller particle size distribution (Figure 2b,d), compared to the sample without zinc.

**Figure 3:**
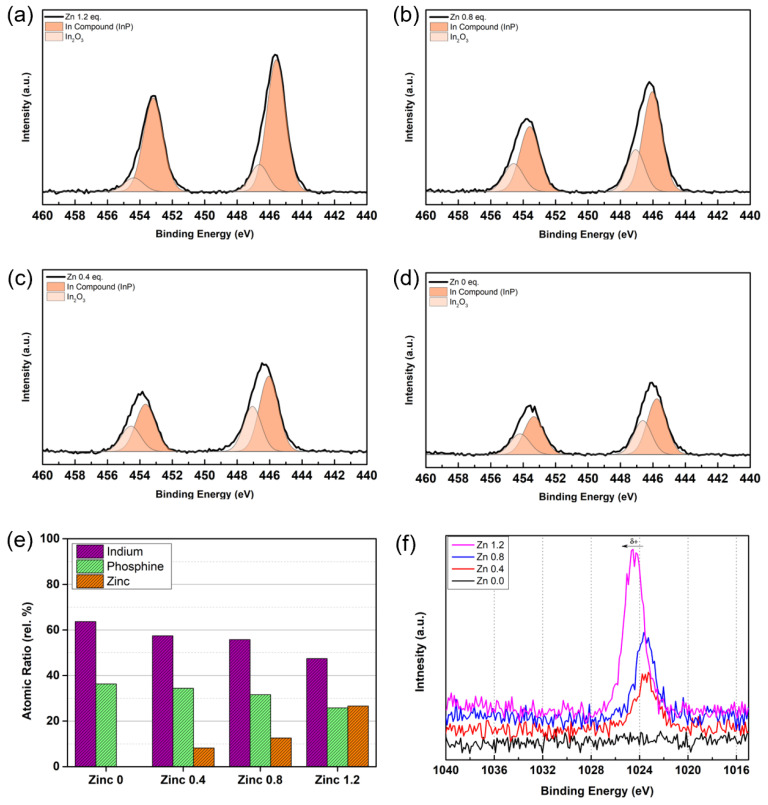
(**a**–**d**) XPS analysis of synthesized samples depending on the Zn loading amounts. (**e**) Quantitative analysis result of the synthesized samples. (**f**) Offset XPS spectrum of Zn 2p.

To assess the effectiveness of the zinc complex passivation, peak deconvolution of the In3d spectrum of each sample was conducted [34]. As the XPS spectrum of the In3d orbital shows a distinctive spin-oriented split, it was able to minimize the error which might be caused by the overlapping orbital split in XPS spectra. As shown in Figure 3a–d, the overall intensity of the indium compound peak has amplified upon the increase of the zinc contents, while the peak assigned to the indium oxide (In_2_O_3_) was diminished. The XPS deconvolution result indicates that the ratio of the oxide layer was decreased upon the increase of the zinc, which supposedly suggests that the zinc complex was able to successfully inhibit the development of the In_2_O_3_ surface oxide layer, preventing degradation of the luminescent quality of the core.

Furthermore, from the Zn2p spectrum of Figure 3f, we can observe the peak of the spectrum was positively shifted as a result of interatomic interaction in-between zinc and phosphorous of the core, which is exposed to the surface and tends to form a bond with the zinc complex. The bond between the II-V compound forms a polar covalent bond toward the V element (phosphorous for the present case), which would induce the positive shift in the binding energy of the zinc spectrum [35]. Inversely, in the case of the In3d spectrum, both peak of the indium spectrum tends to move toward the negative direction when the degree of surficial zinc passivation decreases, which is supposedly resulted of the formation of a negatively charged bond with oxygen, developing In_2_O_3_ layer. Upon this observation, we may conclude that the in situ formed zinc complex was able to successfully passivate the surface of the InP core with an increase in its quantity.

On top of deconvolution and shifted spectrum, the increased intensity of the Zn 2p spectrum implies that the amount of zinc to metal compound (Zn-InP) and zinc oxide was increased at the same time, which indirectly indicates that the zinc complex is successfully located between the outer zinc oxide layer and internal InP core, protecting InP core from the oxidation-oriented deterioration.

## 4. Conclusions

In this work, we investigate the function of the metal carboxylate upon the synthesis of InP CQDs. By utilizing the zinc carboxylate in the synthesis of InP core, the formation of surface In_2_O_3_ layer was successfully suppressed, which was confirmed by XPS analyses. This resulted in an 14-fold increase in PLQY compared with InP core without zinc carboxylate surface passivation. The FWHM also reduced from 130 nm to 70 nm, leading to narrow PL emission spectra. In addition, zinc carboxylate modulated the growth of InP core and resulted in the smaller size of final InP CQDs with 100 nm blue shift of PL emission peak. This result suggests simple and effective way to improve optical properties of III-V CQDs by introducing metal carboxylate complexes.

## Data Availability

Not applicable.
